# Ionic electroactive polymer artificial muscles in space applications

**DOI:** 10.1038/srep06913

**Published:** 2014-11-05

**Authors:** Andres Punning, Kwang J. Kim, Viljar Palmre, Frédéric Vidal, Cédric Plesse, Nicolas Festin, Ali Maziz, Kinji Asaka, Takushi Sugino, Gursel Alici, Geoff Spinks, Gordon Wallace, Indrek Must, Inga Põldsalu, Veiko Vunder, Rauno Temmer, Karl Kruusamäe, Janno Torop, Friedrich Kaasik, Pille Rinne, Urmas Johanson, Anna-Liisa Peikolainen, Tarmo Tamm, Alvo Aabloo

**Affiliations:** 1 Institute of Technology, University of Tartu, Estonia; 2 Active Materials and Smart Living (AMSL) Laboratory, Department of Mechanical Engineering, University of Nevada, Las Vegas, NV, USA; 3 Department of Mechanical Engineering, University of Nevada, Reno, NV, USA; 4 Laboratoire de Physicochimie des Polymères et des Interfaces, Institut des Matèriaux, Université de Cergy-Pontoise, Cergy, France; 5 Health Research Institute, National Institute of Advanced Industrial Science and Technology (AIST), Osaka, Japan; 6 School of Mechanical, Materials and Mechatronic Engineering, University of Wollongong, Australia; 7 ARC Centre of Excellence for Electromaterials Science (ACES), University of Wollongong, Australia

## Abstract

A large-scale effort was carried out to test the performance of seven types of ionic electroactive polymer (IEAP) actuators in space-hazardous environmental factors in laboratory conditions. The results substantiate that the IEAP materials are tolerant to long-term freezing and vacuum environments as well as ionizing Gamma-, X-ray, and UV radiation at the levels corresponding to low Earth orbit (LEO) conditions. The main aim of this material behaviour investigation is to understand and predict device service time for prolonged exposure to space environment.

An essential metric in spacecraft design and optimization is the payload being lifted into space. Large-volume, heavy materials can limit the mission capabilities, whereas lightweight, compact, and unsophisticated energy-effective technologies enable missions to spend more time in space, travel farther, and explore new destinations. Research toward promising space-worthy technologies has approved two principal classes of electroactive polymers: dielectric and ionic^1^,^2^. The class of ionic electroactive polymers (IEAPs) are one of the auspicious candidates, meeting all criteria for the desired technologies^3^,^4^.

During the last three decades, the family of IEAPs has expanded from the primeval soft polyelectrolyte gel^5^ to three principal branches, which do not have clear-cut boundaries: ionic polymer-metal composites (IPMC) with noble metal electrodes^6^,^7^, conducting polymer actuators^8^, and nanocarbon-based ionic actuators^9^. Although the working mechanisms differ, they all have similar configurations: a three-layer laminate composed of an ionic conductive membrane sandwiched between two conductive electrodes. Their distinctive electromechanical bending behaviour is caused by movement of the electrolyte ions between the two opposite faces of the membrane, operating concurrently as an insulator and as a reservoir of the electrolyte. The electrodes have two functions; they provide a large specific surface area to accommodate the moving ions and form a conductive trace to the electrical terminals.

In general, IEAPs are lightweight and soft multi-functional materials, offering significant benefits over traditional technologies. The relatively rigid IEAP laminate can be used concurrently as an active element—actuator or sensor—and a structural member of the device. The IEAP actuators can respond to a low-voltage signal with a large bending stroke without requiring rotating parts, shafts, or precautions against friction. An IEAP actuator can sense motion^10^ or moisture^11^, harvest energy^12^, and store electric energy as a supercapacitor^13^, and the same portion of the laminate can perform several different functions. The unsophisticated structure of the three-layer laminate allows the applications to be downscaled in a natural way using intersection. An IEAP actuator or sensor can easily be miniaturized for microelectromechanical systems or lab-on-a-chip applications with lateral dimensions as small as 50 µm^14^. Due to the prominent features, ample IEAP-based space-worthy devices have been proposed during the last decade^15^,^16^,^17^. An attempt, worth mentioning, to send a piece of IEAP really to space was the MUSES-CN mission^18^. The dust wiper on the infrared camera window was propelled by an ionic polymer-metal composite (IPMC), which was the only well-studied IEAP type at the time the project was proposed. The idea was promising because the operationality of IPMCs under low pressure and low temperature had been previously explored by Shahinpoor et al^19^,^20^,^21^. Unluckily about a year before launch the whole project was cancelled.

In addition to the weight, another critical attribute of space systems is reliability because any reliability problems during the mission can lead to complete mission failure. Therefore, any technology design intended for orbit is carefully inspected for potential causes of failure prior to launch. To demonstrate that IEAP actuators survive the space radiation effects, we performed ground-laboratory tests with seven types of IEAP actuators, covering a wide variety of constituents and fabrication technologies. The collection of the test objects was chosen among well-studied IEAP materials at the present time, such as aqueous IPMC, conducting polymer actuators, and nanocarbon-based ionic actuators. The IEAP materials involved in the experiments are briefly compared in Table 1, while a comprehensive overview of their fabrication and properties is given in the Supplementary Information and in the corresponding references.

This project was in part conducted with support from the European Space Agency (ESA). Therefore, the adverse environments and their levels were chosen to conform to those of the low Earth orbit (LEO). The synergism effects—the simultaneous action of several destructive factors that can enhance or reduce the resulting effects—were not studied. The 50–60 samples of each of the seven IEAP materials were divided into six test groups. Each group was subjected to one of the following conditions:

- **X-ray** radiation with radiation intensity of 139.5 R min^−1^. The X-ray exposure lasted 120 min, yielding a total radiation dose of 167.4 Gy;
- **Gamma** radiation with the ^60^Co radioactive isotope. With the dose rate of 86 Gy h^−1^, the total radiation dose reached 2036 Gy;
- **UV** radiation with a xenon gas-discharge lamp at a distance of 25 cm. The samples were turned over every 24 hours, and the total exposure lasted for 180 hours;
- **Vacuum**ing for two weeks in a vacuum chamber at a pressure less than 1 mbar;
- **Low Temp**erature freezing at the temperature of boiling nitrogen (77 K) for two weeks or at the temperature of boiling helium (4.22 K) for 10–15 minutes;
- **Reference** samples were kept in ambient conditions.

The abbreviations indicated with bold text indicate the corresponding series in the graphs given below. Detailed descriptions of the conditions in harsh environments are given in the Supplementary Information.

## Long-term performance test

The sequence of the experiment consisted of the following steps:

Step 1. The initial performance of each sample was tested during a single measurement cycle;

Step 2. Without operating, the samples were exposed to their pre-assigned hazardous environments;

Step 3. The performance of all samples under continuous loading was recorded until their complete degradation. This step was carried out in an ambient environment (temperature 17°C, relative humidity 30%) after the hazardous environmental exposure was terminated.

Step 3 involves measuring the electrical and electromechanical impedances of the actuators under continuous load. To ensure repeatability of the test procedure, the process was conducted on original large-scale test equipment, which is described elsewhere^31^. This process was terminated when the performance of most of the samples was below the pre-determined threshold: 1% of the average initial performance value. Depending on the IEAP type, complete degradation occurred in 3–25 days, producing an immense amount of recorded measurement readings. The obtained data set allows the electrical and electromechanical impedances of each sample to be tracked throughout its lifetime. The subsequent data analysis revealed that the degradation of any IEAP type could be conveniently evaluated from a single characteristic - performance, which describes the bending ability of a particular actuator at the given moment. The performance parameter that is easily applicable to all situations is the angular spread of the actuator^31^.

**Figure 1:**
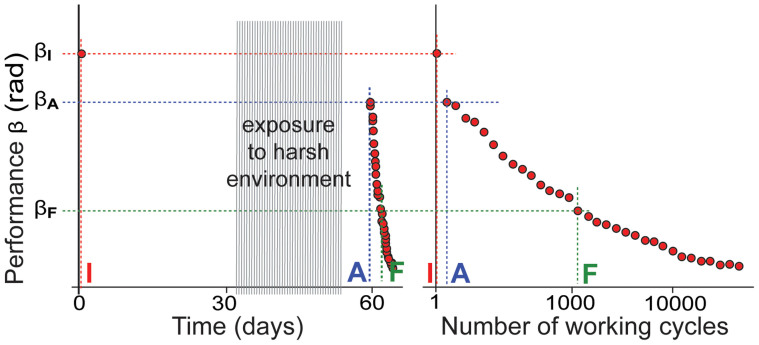
The left graph shows two phases of the lifetime of actuators I-A and A-F and the corresponding performances (angular spreads) β_I_, β_A_, and β_F_, while the right graph shows the number of passed working cycles for the same actuators. Each point in the left graph corresponds one point at the right graph and vice versa.

In the course of the Step 3, performance tests, which lasted 13 working cycles, were performed after every 174 working cycles. The obtained data allows the performance of each sample to be plotted with respect to time as well as the total number of cycles performed. Typical plots with respect to time and the total number of cycles are given in Figure 1. Each point denotes one test-cycle, and one point in one graph corresponds one point in the other graph and vice versa.

## Results

For analysis, the lifetime of an IEAP actuator was divided into two phases, indicated by the three checkpoints in Figure 1: **β_I_** indicates the sample initial performance at the first test **I**, **β_A_** indicates the performance at the first test **A** after the sample is removed from the adverse environment; and **β_F_** indicates the final performance after an arbitrary number of working cycles **F** before remarkable degradation begins. Due to the fact that the lifetimes of different IEAP types may vary up to several orders of magnitude, the checkpoint **F** is chosen separately for each particular IEAP type.

**Figure 2:**
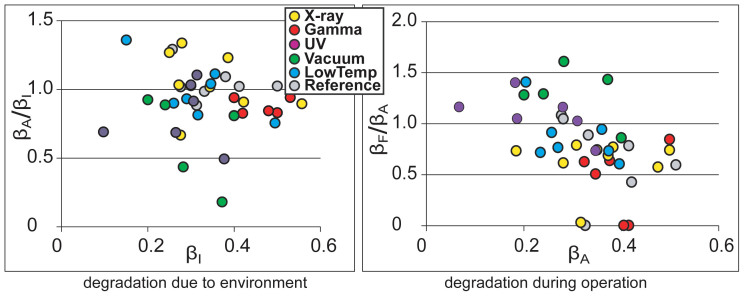
IEAP material *A*: a traditional IPMC with a perfluorinated sulfonic acid ionomer membrane and electrodes made of chemically plated platinum with a palladium supporting layer^22^,^23^. This IEAP material contains water and is intended to work in an aqueous environment. Checkpoint **F** is marked at 10,000 working cycles. Drying in vacuum and subsequent regeneration slightly affects the performance of this IEAP material. All other environmental parameters have no notable effect.

**Figure 3:**
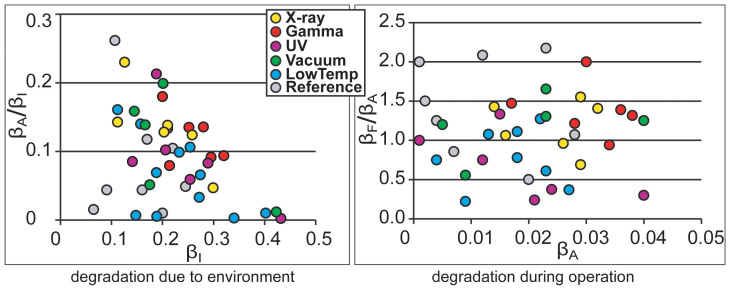
IEAP material *B*: A non-ionic polymer membrane with bucky-gel electrodes. A PVdF-HFP polymer membrane with electrodes made of bucky-gel, which is a gelatinous mixture of single-walled carbon nanotubes (SWCNT) and ionic liquid, particularly EMIBF_4_^24^,^25^. Checkpoint **F** is marked at 3000 working cycles. Degradation due to environment is perceivable, but in spite of the considerable divergence of the initial performance, none of the environmental parameters has notable effect on this IEAP material.

**Figure 4:**
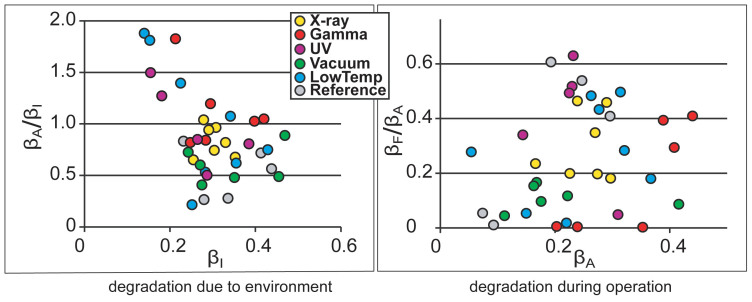
IEAP material *C:* An ionic polymer membrane with nanoporous carbon electrodes. A Nafion® membrane with electrodes consisting of CDC, which is nanoporous carbon derived from titanium carbide^33^. The electrolyte is ionic liquid (IL), 1-ethyl-3-methylimidazolium trifluoromethanesulfonate (EMITF), while the conductivity of the electrodes is improved by thin gold foil^26^. Checkpoint **F** is marked at 3000 working cycles. In spite of the considerable divergence of the initial performance, none of the environmental parameters has a notable effect on this IEAP material.

**Figure 5:**
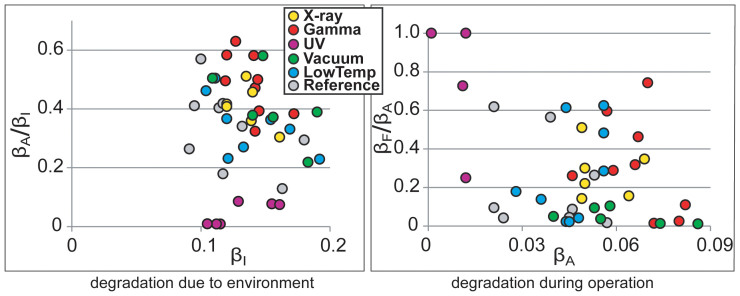
IEAP material *D*: A non-ionic polymer (PVdF-HFP) membrane with CDC electrodes and electrolyte EMIBF_4_^27^. Checkpoint **F** is marked at 5000 working cycles. Exposure to UV radiation significantly damages this IEAP material, although it is able to function with lower performance. Vacuuming increases the degradation during operation.

**Figure 6:**
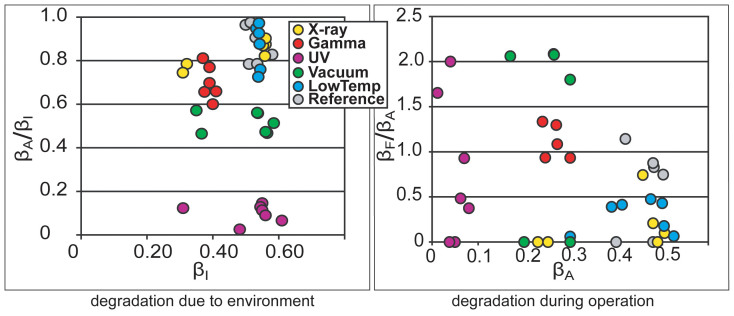
IEAP material *E*: a conducting interpenetrating polymer network, based on a non-homogeneous dispersion of PEDOT through the thickness of the PEO/NBR IPN matrix, with EMITFSI as electrolyte^28^. This material is similar to a layered actuator with conducting polymer electrodes with the advantage that no adhesive interface is necessary. Checkpoint **F** is marked at 10,000 working cycles. The samples were fabricated in two batches; therefore, the initial performances lie in two intervals. UV radiation destroys this IEAP material. Indeed, the photo-oxidation process leads to rapid degradation of the polyethylene oxide (PEO) partner, which in turn degrades the PEDOT electrodes. All other environmental parameters have no notable effect. Vacuuming slightly degrades this IEAP material; however later the degradation is not noticeable.

**Figure 7:**
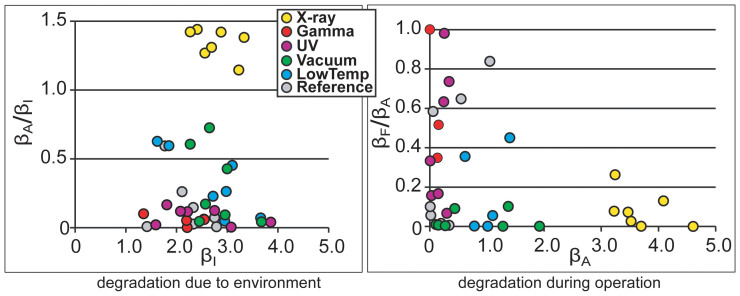
IEAP material *F*: Ppy films grown galvanostatically on gold-coated PVdF membrane. The electrolyte is 0.1 M LiTFSI solution in PC^29^. Checkpoint **F** is marked at 600 working cycles. The degradation due to the environment is perceivable. Direct UV radiation certainly destroys this IEAP material. Moreover, X-ray radiation increases the performance significantly; however, this effect only lasts a few measurement cycles.

**Figure 8:**
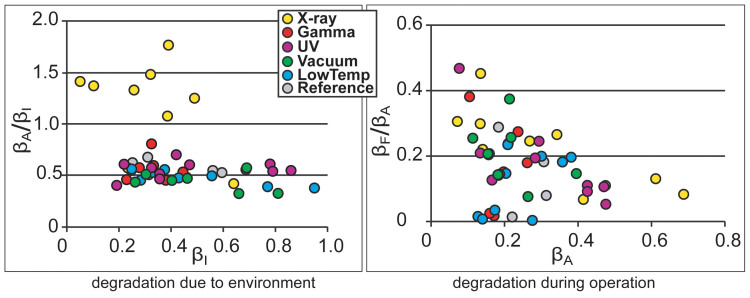
IEAP material *G*: a conducting polymer IEAP comprising PVdF membrane with Ppy electrodes and PC+LiTFSI (1.0 M) electrolyte fabricated by the combined chemical and electrochemical synthesis method^30^. Checkpoint **F** is marked at 500 working cycles. The degradation due to environment is much less noticeable than the previous case (***F***). The influence of UV is not noticeable. After exposure to X-ray radiation, the performance is significantly increased; however, the effect only lasts a few measurement cycles.

The long-term degradation experiment was carried out using identical methodology on 320 IEAP actuators of seven types, divided into six test groups. The subsequent survival analysis of the actuators confirmed the deduction of Liu et al. – the distribution of lifetimes cannot be well described by any of the commonly used methodologies of failure distribution characterization, e.g. the Weibull statistics^32^. Moreover, with small statistical sample sizes (up to 10 samples of each category) of the rather irregular test objects, the formal statistics also become uncertain. Therefore, we explore the common degradation tendencies using visual data analysis methods. The obtained data is displayed graphically by scatter plot pairs, which present the dimensionless relationships between the initial performance and final performance of the two phases. Along the horizontal axes of the two scatter diagrams are plotted the starting performances of the corresponding phases, **β_I_** and **β_A_**. Along the vertical axes are plotted the degradation due to the environment (**β_A_/β_I_**) and the degradation during operation (**β_F_/β_A_**) respectively. The seven scatter plot pairs presented in Figures 2,3,4,5,6,7,8 describe the degradation tendencies of all 320 samples of seven IEAP types.

## Discussion

Though X-ray radiation significantly increases the performance of the Ppy actuators, the higher performance is indiscernible by the next measurement cycle. On the other hand, Gamma radiation had no effect on IEAP materials with conducting polymer electrodes (***F*** and ***G***). This indicates that the interim performance peak is due to the radiation-induced doping of conducting polymer electrodes, which improves their conductivity. The described phenomenon has not yet been studied in detail. Furthermore, the few available studies about the ionizing radiation-induced doping of conductive polymers do not cover our specific case^34^,^35^. An intelligible explanation to the phenomenon of ionizing radiation-induced doping is given by Boye et al.^36^ At lower doses, the ionizing radiation acts as a catalyst, allowing excess dopant to attach to the polymer chain. This, in turn, enhances the material conductivity. As the dose increases, damage to the polymer chains will be the dominant result of ionizing radiation, leading to a further decrease of conductivity. However, all studies on this topic deals with a separate conducting polymer. In our experiment, the alternate polarization of the actuator during the next working cycle nullifies the effect of excess dopants.

## Conclusions

This study confirms that cosmic radiation does not impinge on the prolonged exploitation of IEAP actuators in space applications. Unlike materials used for conventional actuators, IEAP materials do not embody any crystal lattice, the characteristics of which could be sensitive to ionizing radiation or atomic oxygen. Instead, these laminates consist of polymeric materials with high-radiation resistance: PVdF, Nafion, carbon powder, noncrystalline noble metals, and nonvolatile ionic liquid electrolytes. Although numerous methods exist to modify the structure of these materials with ionizing radiation, the damaging doses are several magnitudes higher than any object can absorb from cosmic radiation in space near Earth over several decades^37^,^38^.

The results of our long-term large-scale experiment convincingly demonstrate that IEAP actuators are fully tolerant to the ionizing Gamma- and X-ray radiation at LEO levels. The IEAP actuators with carbonaceous electrodes as well as aqueous IPMC are resistant to all environmental parameters tested. The most destructive radiation for IEAP actuators is direct UV; hence, devices obscured from direct sunlight can be considered to be fully reliable. Moreover, UV-degradation of the conducting polymers PEDOT and Ppy is described in numerous papers^39^,^40^; it also caused harm to the radiation-absorbing black electrodes of IEAP material D. Long-term vacuuming does not affect actuators that have ionic liquid electrolytes. The freezing temperature and duration show no difference. Naturally, these materials are not able to work when the electrolyte is frozen or drawn off, but they revive after melting up or soaking in the appropriate electrolyte.

## Methods

The experiment involved testing 320 samples. Over the course of the long-lasting experiment, the electrical input and electromechanical output were recorded for each sample. To handle the number of samples, we designed original equipment to automatically perform the testing procedure upon many actuators concurrently. A comprehensive overview of the equipment as well as the methodology used for testing is presented elsewhere^31^. This setup excludes human errors and guarantees that all samples are tested under exactly similar conditions. For that reason, the experiment was setup considering the trade-up between the obtained data and the automation options.

**Figure 9:**
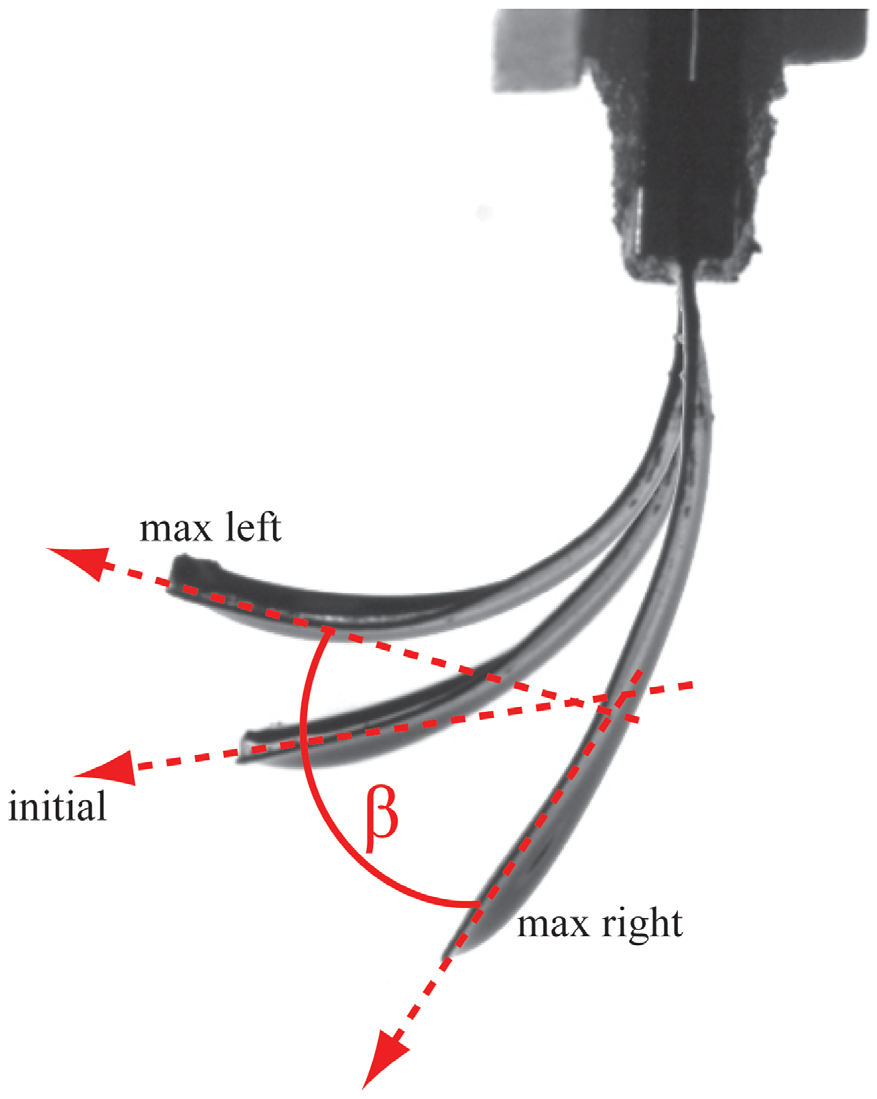
The performance of an IEAP actuator is defined as β, which is the angular spread of the tangent of the actuator tip.

The recorded mechanical outputs were the force gauge output and visual information on the bending. The actuator behaviour was recorded by a camera, which was equipped with a long-focal-length lens. The 640 × 480 pixel images were converted to vector interpretation using the National Instruments LabView image processing package. The vectorial interpretation expresses the curvature as a function of the distance from the input contacts along the sample. The curved line representing the shape of the actuator is divided into vectors of equal length, assuming that the curvature is constant within each vector^41^. The shape of the actuator is characterized by the matrix of angles of each vector with respect to the direction of the previous vector. A convenient quantitative parameter for estimating the performance of the actuator is the angle β between the tangents of the tip of the actuator in the case where the maximal bending displacements are in opposite directions, as depicted in Figure 9. This parameter is valid until the actuator bends in the camera's field of view, and this parameter adequately reflects the performance even in the case of considerable initial creep of the sample.

The recorded electrical signals involved the input voltage and the input electric current, measured as a voltage drop over a shunt resistor of a properly chosen value. The exciting voltage signal during a single measurement cycle was a gradual sweep. The exciting voltage consisted of series sequence of sine signals of nine different frequencies: 10, 5, 2.5, 1.25, 0.64, 0.32, 0.16, 0.08, and 0.04 Hz. To distinguish the two types of degradation, the experiment was accelerated by choosing the amplitude of the driving voltage close to the uppermost allowed voltage for each IEAP type. The obtained data set allows tracking of the electrical and electromechanical impedances for each sample throughout its lifetime. In the scope of the current report, we show only the performance behaviour (angle between the tangents of the actuator tip where the maximal bending displacements are in opposite directions) at the lowest frequency of 0.04 Hz.

## Supplementary Material

Supplementary Information Supplementary Information

## Figures and Tables

**Table 1:** IEAP materials

	Membrane	Electrodes	Electrolyte	References
**A**	GEFC	Pd+Pt	Water+Li^+^	22,23
**B**	PVdF-HFP	SWCNT	EMIBF_4_	24,25
**C**	Nafion®	nanoporous carbon+Au	EMITF	26
**D**	PVdF-HFP	nanoporous carbon	EMIBF_4_	27
**E**	PEO/NBR IPN	PEDOT	EMITFSI	28
**F**	PVdF	Ppy +Au	PC+ LiTFSI	29
**G**	PVdF	Ppy	PC+ LiTFSI	30

Acronyms: EMIBF_4_: 1-ethyl-3-methylimidazolium tetrafluoroborate; EMITF: 1-ethyl-3-methylimidazolium trifluoromethanesulfonate; EMITFSI: ethyl-3-methylimidazolium bis-(trifluoromethylsulfonyl)imide; GEFC: perfluorinated sulfonic acid ionomer of GEFC Co., Ltd; LiTFSI: lithium bis(trifluoromethane)-sulfonimide; Nafion®: perfluorinated anionic polymer of DuPont; PC: propylene carbonate; PEDOT: poly(3,4-ethylenedioxythiophene); PEO/NBR IPN: polyethylene oxide/nitrile butadiene rubber interpenatrating polymer network; Ppy: polypyrrole; PVdF: poly-1,1-difluoroethene, polyvinylidene fluoride; PVdF-HFP: poly(vinylidene fluoride-co-hexafluoropropene); SWCNT: single-walled carbon nanotubes.
