# Supplementary material for: Ionic electroactive polymer artificial muscles in space applications
Source: Sci Rep. 2014 Nov 5;4:6913. doi: 10.1038/srep06913 (PMC5381380; doi:10.1038/srep06913)
Supplement: Supplementary Information [file srep06913-s1.pdf]

Supplementary Information

Ionic electroactive polymer artificial muscles in space applications

Andres Punning*, Kwang J. Kim, Viljar Palmre, Frédéric Vidal, Cédric Plesse, Nicolas Festin, Ali Maziz, Kinji Asaka, Takushi Sugino, Gursel Alici, Geoff Spinks, Gordon Wallace, Indrek Must, Inga Põldsalu, Veiko Vunder, Rauno Temmer, Karl Kruusamäe, Janno Torop, Friedrich Kaasik, Pille Rinne, Urmas Johanson, Anna-Liisa Peikolainen, Tarmo Tamm, Alvo Aabloo

* corresponding author, e-mail: andres.punning@ut.ee

Acronyms and abbreviations:

- CDC – titanium carbide – derived carbon;
- DMAc – N,N-dimethylacetamide;
- EDOT – 3,4-ethylenedioxythiophene;
- $EMIBF_4$ – 1-Ethyl-3-methylimidazolium tetrafluoroborate;
- EMITF – 1-ethyl-3-methylimidazolium trifluoromethanesulfonate;
- EMITFSI – ethyl-3-methylimidazolium bis-(trifluoromethylsulfonyl)imide;
- GEFC – perfluorinated anionic polymer of GEFC Co., Ltd;
- IEAP – ionic electroactive polymer;
- IL – ionic liquid;
- IPMC – ionic polymer-metal composite;
- IPN – conducting interpenetrating polymer network, an intimate combination of two cross-linked polymers;
- LiTFSI – lithium bis(trifluoromethane)-sulfonimide;
- MEG – monoethylene glycol;
- NaDBS – sodium dodecylbenzenesulfonate;
- Nafion® - perfluorinated anionic polymer of DuPont;
- $Na_2S_2O_8$ – Sodium peroxodisulfate;
- NBR – Nitrile Butadiene Rubber
- PC – propylene carbonate (4-methyl-1,3-dioxolan-2-one);
- PEDOT – poly(3,4-ethylenedioxythiophene);
- PEGM – methoxy poly(ethylene glycol) methacrylate;
- PEGDM – poly(ethylene glycol) dimethacrylate;
- PEO/NBR IPN – polyethylene oxide / nitrile butadiene rubber interpenetrating polymer network;
- PVdF – poly-1,1-difluoroethene, polyvinylidene fluoride;
- PVdF(HFP) – poly(vinylidene fluoride-co-hexafluoropropylene);
- Py – pyrrole;
- Ppy – polypyrrole;
- SWCNT – single-walled carbon nanotubes.

IEAP materials, their preparation, and notes.

A. A water-based IPMC with perfluorinated sulfonic acid ionomer membrane (GEFC Co., Ltd) and electrodes made of chemically plated platinum with palladium supporting layer. This IEAP material contains water, and it is intended to work mainly in water. For measurements in air, the samples were lifted out of water for about 130 seconds only.

Preparation: The pretreated water-containing ionomer membrane was plated with Pd using a chemical reduction reaction from tetraamminepalladium(II) chloride monohydrate. In order to increase the surface conductivity, an additional Pt layer from tetraammineplatinum(II) chloride monohydrate was chemically deposited on top of the palladium surface. The Li^+ cations were introduced by soaking the metallized film in 1M solution of LiCl.

Fabricated in the University of Nevada, Reno, USA. For the details, see [22,23].

B. A PVdF(HFP) membrane with electrodes made of a gelatinous mixture of SWCNT and IL – i.e. the bucky-gel. $EMIBF_4$ was used as IL.

Preparation: The actuator is obtained by hot-pressing the electrode and electrolyte layers. Both layers are prepared by casting the appropriate solution or mixture and subsequent evaporation of the solvents. The bucky-gel electrode mixture is composed of SWCNTs, $EMIBF_4$ and solution of PVdF(HFP) in DMAc. The electrolyte layer is composed of $EMIBF_4$ and PVdF(HFP) solution in DMAc.

Fabricated in the National Institute of Advanced Industrial Science and Technology (AIST), Japan. For details, see [24,25].

C. A nafion membrane with electrodes consisting of nanoporous carbide–derived carbon. The electrolyte is EMITF IL, while the conductivity is improved by thin gold foil.

Preparation: pretreated Nafion membrane is immersed in ionic liquid - (EMITF). The mixture for the carbon electrodes consisted of CDC powder, Nafion dispersion in water and isopropanol. 10-15 layers of the conductor/ionomer mixture were applied to the membrane using an airbrush. Volatile solvents were removed under an infrared lamp after application of each layer. Finally, the membranes were sandwiched between two gold foils (270 nm thick) and fused together by hot-pressing. A layer of 5% Nafion solution was used to provide better adhesion to the gold foil.

Fabricated in the University of Tartu, Estonia. For the details, see [26].

D. A PVdF(HFP) membrane with electrodes consisting of nanoporous carbide–derived carbon. The electrolyte is $EMIBF_4$ ionic liquid.

Preparation: The electrode layer, composed of CDC powder, $EMIBF_4$, and PVdF(HFP) was made by casting the suspension into a Teflon mold and then drying at 80° C in a vacuum oven. The electrolyte film was obtained by pouring the mixture of PVdF(HFP) and $EMIBF_4$ into a Teflon mold and evaporating the solvent completely. Finally, the electrolyte film was sandwiched between two electrode films and fused together by hot-pressing.

Fabricated in the University of Tartu, Estonia. For the details, see [27].

E. A conducting interpenetrating polymer network (C-IPN) based on non-homogeneous dispersion of PEDOT through the thickness of the PEO/NBR IPN matrix with EMITFSI as electrolyte. This material is similar to a layered actuator with conducting polymer electrodes, with the advantage that no adhesive interface is necessary. The IPN structure ensures a good compatibility between PEDOT and the matrix, which completely prevents delamination.

Preparation: reactive mixtures are prepared from PEO and NBR precursors. The given amounts of NBR, PEGM, and PEGDM were poured into a glass mould and heated, to yield a PEO/NBR IPN film. The electrodes were applied by soaking the IPN film in pure EDOT. The swollen films were then immersed for 20 h in a $FeCl_3$ aqueous solution (1.5 mol L^{-1}). The film was washed with methanol until the excess of $FeCl_3$ was removed. The conducting IPN films were dried at 60 °C under vacuum for 24 h.

Fabricated in the Université de Cergy-Pontoise, France. For the details, see [28].

F. Polypyrrole doped with $TFSI^-$ ion films grown galvanostatically on a gold-coated PVdF membrane with PC+LiTFSI (0.1M) electrolyte. This IEAP material is capable working in air, but in order to avoid drying, it is recommended to soak it periodically in the electrolyte solution. In the course of the long experiment, the delay between moistening procedures was 110 minutes.

Preparation: Both sides of a 110-µm commercial Millipore PVdF membrane were coated with a thin gold layer using a magnetron sputter coater. Ppy layers were electrodeposited on the conductive surface in a solution containing 0.1 M Py, dissolved in 0.1 M LiTFSI/PC (with 1% water). After polymerization, the actuators were rinsed with acetone and soaked with the 0.1 M LiTFSI/PC solution.

Fabricated in the University of Wollongong, Australia. For the details, see [29].

G. A PVdF membrane with polypyrrole electrodes and with PC+LiTFSI (1.0M) electrolyte, fabricated by the combined chemical and electrochemical synthesis method. This IEAP material is capable working in air, but in order to avoid drying, it is recommended to soak it periodically in the electrolyte solution. In the course of the long experiment, the delay between moistening procedures was 110 minutes.

Preparation: Commercial Millipore PVdF membrane (according to specification: hydrophobic, thickness 125 µm, pore size 0.45 µm, porosity 70%) was used as electrode storage layer. Electrodes for electrochemical synthesis were synthesized chemically as follows: membrane was permeated with pyrrole monomer and immersed in 0.006 M NaDBS, 0.075 M Na2S2O8 aqueous solution at 60 °C for 45 s; the polymerizing Py from outer-most pores turned the membrane black. The chemical synthesis was terminated by washing membrane with cold methanol. PPy was deposited galvanostatically on both sides of the membrane. Electrochemical synthesis was carried out galvanostatically in an one-compartment two-electrode electrochemical cell. The synthesis solution contained 0.2 M Py and 0.2 M NaDBS dissolved in a mix of water and MEG. 1 M LiTFSI in PC was used as actuation electrolyte.

Fabricated in the University of Tartu, Estonia. For the details, see [30]).

Harsh environments

a) **X-ray** radiation was carried out in the Faxitron RX-650 cabinet X-ray system. With the energy of 110 kV, the intensity of radiation was 139.5 R min^{-1}. The exposure lasted 120 min, yielding the total radiation dose of 167.4 Gy. In order to avoid contamination of the radiation chamber, each sample under test was individually encapsulated in a 2-cm^3 glass vial.

b) The **Gamma** radiation test was performed at the ESTEC Co-60 Facility, Noordwijk, Netherlands. The samples ordained to Gamma radiation were divided into 3 groups, exposed to different doses of radiation. Each sample was individually encapsulated into a poly(methyl methacrylate) box unit. Temperature: 25.4 °C; Atmospheric pressure 1007.6 mbar; Relative humidity 44.3%

The doses and dose rates were:

	Group 1	Group 2	Group 3
Total Ionising Dose (Gy)	400.25	1206.00	2035.90
Dose Rate (Gy h^{-1})	73.56	73.65	86.56

However, we did not notice any difference between the three groups in the case of all seven IEAP materials.

The repeat test was carried out at Steri – an Estonian company, providing irradiation services with the Co-60 source. The dose rate was 1180 Gy h^{-1} and the total ionizing dose was 2180 Gy. Each sample under test was individually encapsulated in a 2-cm^3 glass vial.

c) The **UV** radiation was carried out in the Institute of Physics, University of Tartu. The source of the UV radiation was a xenon gas-discharge lamp at the distance of 25 cm. The samples were exposed directly to the UV source at the distance of 25 cm. At this distance, the air temperature measured close to the surface of the samples was 37 °C. Periodically, but not more often than in 24 hours, the samples were turned over in order to expose the other side. In this regime, both sides of all samples were exposed to the source for 178 hours and 42 minutes.

Due to the elevated temperature, the IEAP actuators of types D, E, F, and G occurred being dried out. Therefore, they were soaked in the appropriate solvent for at least 2 days before performing the lifetime test.

d) Study of **temperature** effects

Two different tests regarding the temperature effects were carried out in the University of Tartu:

1. Short-term freezing to the temperature of boiling helium (4.22K) for at least 10 minutes;
2. Keeping the samples at the temperature of boiling nitrogen (77K) for 60 days.

The samples were individually encapsulated in closed 2-ml high-grade polypropylene Greiner bio-one Cryo-s vials. The melt-up was carried out in the room temperature during at least 24 hours, while the samples were still in their vials. The latter analysis did not show any difference between these experiments, therefore the corresponding samples were grouped together.

e) **Vacuum.** The samples were held in vacuum chamber under pressure of <1 mb for two weeks. The experiments were conducted in the University of Tartu.

The IEAP actuators of types D, E, F, and G dried out. Therefore, they were soaked in the appropriate solvent for at least 2 days before performing the lifetime test.
